# Association between regional dietary patterns and cardiovascular health status among elderly in China

**DOI:** 10.21203/rs.3.rs-2328623/v1

**Published:** 2023-01-18

**Authors:** Yingying Jiao, Weiyi Li, Xiaofang Jia, Zhihong Wang, Huijun Wang, Bing Zhang, Hongru Jiang, Gangqiang Ding

**Affiliations:** Chinese Center for Disease Control and Prevention; Chinese Center for Disease Control and Prevention; Chinese Center for Disease Control and Prevention; National Institute for Nutrition and Health, Chinese Center for Disease Control and Prevention; Chinese Center for Disease Control and Prevention; National Institute for Nutrition and Health, Chinese Center for Disease Control and Prevention; Chinese Center for Disease Control and Prevention

**Keywords:** elderly, dietary patterns, cardiovascular health, Life’s Essential 8

## Abstract

**Objectives:**

To evaluate the cardiovascular health (CVH) status of the elderly and analyze the effects of dietary patterns and demographic characteristics on CVH.

**Methods:**

A total of 4299 individuals aged 60 years and above from the China Health and Nutrition Survey in 2018 were selected as the research objects. Cluster analysis was used to analyze the dietary patterns. The definition of “Life’s Essential 8” of CVH released by American Heart Association (AHA)in 2022 was used to evaluate CVH status. Finally, multinomial logit model was used to analyze the impact of demographic economic characteristics on CVH.

**Results:**

Three dietary patterns were obtained by cluster analysis. In pattern 1, the intake of wheat, other grains, tubers and legumes was higher. Pattern 2 was dominated by high intake of aquatic products, vegetables and fruits; Pattern 3 was dominated by higher intake of rice and livestock meat. The total CVH score was 68.50, and sleep and blood pressure had the highest and lowest scores (85.85 and 37.64). Pattern 1 and Pattern 2 have slightly higher CVH scores. There were 16%–18% of the elderly with high CVH, and there was no significant difference in the distribution of high, moderate and low CVH among the three patterns (p=0.29). More than 50% of the elderly have 3–4 ideal metrics, 0.2% of the elderly have all 8 metrics reached the ideal state only in pattern 1. Multinomial logit analysis showed that the elderly in pattern 2 had 6–8 ideal metrics, which was 1.81 times higher than that in pattern 1; The presence of 6–8 ideal metrics in female was 3.42 times higher than that in male; Those with a college degree and above have 6–8 ideal metrics, which was 1.99 times of those with a primary school degree and below. Compared with 60–69 years, the presence of 6–8 ideal metrics in 70 years and above was 35% lower (OR=0.65,95%=0.49–0.87). The presence of 6–8 ideal metrics in high income group were 31% lower than those in low income group (OR=0.69,95%=0.47–1.00).

**Conclusions:**

The elderly in China were in moderate CVH. Dietary pattern characterized by higher intake of aquatic products, vegetables and fruits were more likely to have more ideal CVH metrics. It is necessary to take targeted intervention measures for the elderly and health factors with low scores to promote the improvement of CVH status.

## 1. Introduction

Cardiovascular diseases(CVD) are the leading cause of death and disability worldwide. Global burden of disease showed that the number of people with CVD increased from 271 million in 1990 to 523 million in 2019 [1]. With the aggravating aging population in China, the incidence of CVD continued to rise, accounting for more than 40% of disease deaths in Chinese residents [2], ranking first among the total causes of death among urban and rural residents, 46.74% in rural areas and 44.26% in urban areas [3], bringing increasingly heavier economic burden to residents and society. At present, the internationally recognized modifiable risk factors for CVD are hypertension, diet and dyslipidemia, etc. [1]. In addition, the clustering of the above risk factors was more likely to cause the CVD than a single factor [4]. In order to promote the overall improvement of cardiovascular health (CVH), the American Heart Association (AHA) in 2022 proposed the “Life’s Essential 8”, including four health behaviors (diet, physical activity, nicotine exposure and sleep) and four health factors (BMI, cholesterol, blood glucose and blood pressure) [5], which added sleep on the basis of the “Life’s Simple 7 (LS7)” proposed in 2010, and adjusted the quantification of metrics [6].

Studies have found that a good dietary pattern could reduce the incidence and mortality of CVD, diabetes, hypertension and other chronic diseases [7, 8], and most diet-related deaths were caused by CVD, T2D and cancer [9]. Good dietary pattern has become the basis for the current treatment of CVD, as well as an extremely important link in the primary prevention of this disease [10]. As the main population of CVD, the related risk factors of the elderly have been paid more and more attention. It is particularly important to move forward the prevention window of CVD. Therefore, this study used the data from the 2018 China Health and Nutrition Survey (CHNS) to evaluate the CVH status of the elderly under different regional dietary patterns in 15 provinces, so as to provide a scientific basis for appropriate intervention measures.

## 2. Materials And Methods

### 2.1 Study Design and Subjects

This study used data from CHNS, which started in 1989 and conducted in 15 provinces: Heilongjiang (started in 1997), Shandong, Henan, Guangxi, Liaoning (not in 1997), Jiangsu, Hubei, Hunan, Guizhou, Beijing, Shanghai, Chongqing, Shaanxi, Yunnan and Zhejiang. In 2011, three municipalities (Beijing, Shanghai and Chongqing) were added, and in 2015, three provinces (Shaanxi, Yunnan and Zhejiang) were added. By 2018, 11 waves of follow-up had been completed. A stratified multistage cluster random sampling was used to investigate the dietary structure, nutritional status, lifestyle, etc. The same households and members were tracked as far as possible in each round of survey. Specific survey content and sampling scheme refer to literature [11–13]. This study selected people aged 60 years and above as subjects. We excluded those with missing demographic data(n = 870), with missing physical measurement data(n = 528), with missing blood biochemical data(n = 514) ,with missing lifestyle and dietary data(n = 38), and a total of 4229 individuals were finally included in the study.

### 2.2 Dietary pattern analysis

#### 2.2.1 Dietary Survey Methods

Three consecutive 24-hour dietary recalls was used to collect personal food consumption data, and the edible oil and condiments were collected by the household weighing method and allocated to individuals according to the ratio of individual energy consumption in the household, and China Food Composition Table was used to convert the collected consumption of various foods, edible oils and condiments into the intake of various nutrients [14].

#### 2.2.2 Clustering analysis

Cluster analysis was used to reflect the characteristics of differentiated regional dietary patterns. The clustering sample was 15 provinces of CHNS. According to the China Food Composition Table and the dietary habits of the residents, 15 food groups were selected, including wheat, rice, other cereals, tubers, beans, vegetables, fruits, fungus, livestock meat, poultry meat, aquatic products, milk, eggs, nuts, snacks and desserts.

First, the 15 provinces were regarded as 15 separate classes, and then the distance between classes was calculated. The two close classes or several classes were merged into a new class, and the distance between the new class and other classes was calculated. Then the two closest classes or several classes were selected and merged into a new class until all the samples were merged into one class. In this study, the method to determine the distance between the new class and other classes was Ward’s method [15].

### 2.3 Life’s Essential 8” of CVH

#### 2.3.1 Main metrics

A total of 8 metrics, 4 health behaviors (diet, physical activity, nicotine exposure, and sleep) and 4 health factors (BMI, non-HDL-C, blood glucose, and blood pressure). The dietary score was calculated by DASH score [16], and a total of 9 metrics (saturated fat, total fat, protein, cholesterol, fiber, magnesium, calcium, potassium and sodium) were included; Physical activity included leisure, traffic, occupational and household physical activity, and the time of moderate and high intensity physical activity per week was calculated [17]; Nicotine exposure included whether you were a former or current smoker and the duration of smoking cessation; Sleep was the amount of sleep you get each night; The division criteria of BMI made corresponding adjustment according to the criterion of our country [18]. Non-HDL-C was calculated by subtracting HDL-C from TC [19]. Blood glucose was divided by FBG, HbA1 c and whether they had a history of diabetes; Blood pressure was classified by diastolic blood pressure, systolic blood pressure and whether they were on medication.

#### 2.3.2 Calculation of scores

First, the individual score of 8 metrics was calculated, and the full score of each metric was 100 points. Then, the average score was calculated. According to the average score, CVH was divided into three categories: low CVH: <50; moderate CVH:50–79; high CVH:≥80. In addition, metrics with a score of 100 were defined as “ideal metric” in this study, and the specific score calculation was shown in Table 1.

### 2.4 Demographic characteristics

The demographic information involved in this study was obtained by face-to-face survey using special questionnaires by investigators who were uniformly trained and qualified. It mainly includes age, gender, education level, income level and urban and rural areas. The age was divided into two groups(60–69 years, 70 years and above); The education level was divided into three groups: low (primary school and below), middle (middle and high school) and high (college and above); The income was divided into three groups: low (< 10459.87 yuan), middle (10459.87–28718.43 yuan), high (≥ 28718.43 yuan) according to the tertiles of annual household income.

### 2.5 Statistical Analysis

Quantitative variables were expressed as mean ± standard deviation, and categorical variables were expressed as percentage (%). The dietary patterns of different regions were analyzed by clustering analysis. ANOVA was used to compare differences in food consumption and metric scores between groups. Dwass-Steel-Critchlow-Fligner (DSCF) method was used for pairwise comparison among the three groups. Chi-square test was used to compare CVH distribution between different groups. Multinomial logit model was used to analyze the impact of demographic economic characteristics on CVH. All data were analyzed using SAS (Version 9.4, SAS Institute Inc., NC) and we defined statistical significance as p < 0.05.

## 3. Results

### 3.1 Dietary patterns and characteristics of food intake

**Figure 1:**
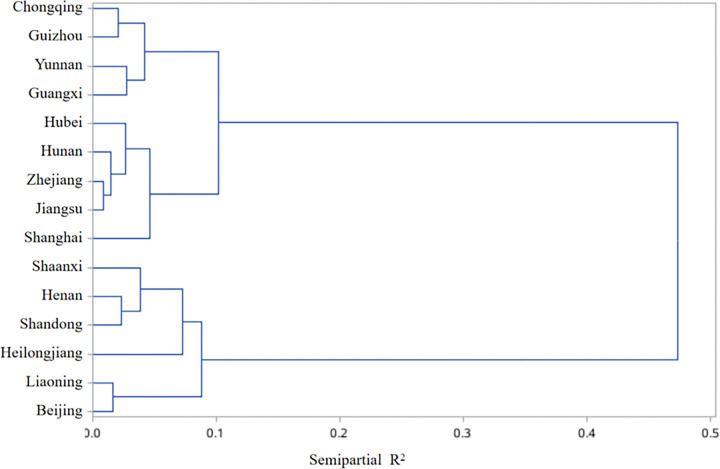
Cluster analysis of dietary patterns. Three dietary patterns were obtained. Pattern 1 included Beijing, Liaoning, Heilongjiang, Shandong, Henan and Shaanxi; Pattern 2 included Zhejiang, Jiangsu, Shanghai, Hunan and Hubei; Pattern 3 included Guangxi, Yunnan, Guizhou and Chongqing.

A total of 4229 subjects were included in this study, of which 62.50%, 46.72% and 37.46% were 60–69 years, male and urban residents, respectively (Supplementary Table 1). According to the characteristics of food intake in different regions, cluster analysis showed that there were three dietary patterns (Fig. 1). Pattern 1 was dominated by northern provinces (Beijing, Liaoning,Heilongjiang, Shandong, Henan and Shaanxi) with higher intakes of wheat, other grains, tubers and legumes; Pattern 2 was dominated by coastal areas and southern provinces (Zhejiang, Jiangsu, Shanghai, Hunan and Hubei), with high intake of aquatic products, vegetables and fruits; Pattern 3 was dominated by southwest provinces (Guangxi, Yunnan, Guizhou and Chongqing), where the intake of rice, livestock and poultry meat was higher. (Table 2).

### 3.2 Cardiovascular health in different dietary patterns

#### 3.2.1 The score of each metric

The total CVH score was 68.50. Among the eight metrics, the scores of sleep and physical activity were relatively high (85.85 and 85.04, respectively). The scores of diet and blood pressure were lower (46.33 and 37.64, respectively). Pattern 1 and pattern 2 had slightly higher overall scores. Analysis of individual metric showed that pattern 2 had higher scores for BMI(no significant difference from pattern 3), non-HDL-C, blood glucose, sleep (no significant difference from pattern 1) and physical activity. Dietary scores in pattern 1 were higher; Pattern 3 had higher blood pressure score. Analysis of demographic characteristics found that the 60–69 years, female, higher education scored relatively high on most metrics. (Table 3)

#### 3.2.2 Distributions of absolute point scores for each CVH metric

**Figure 2:**
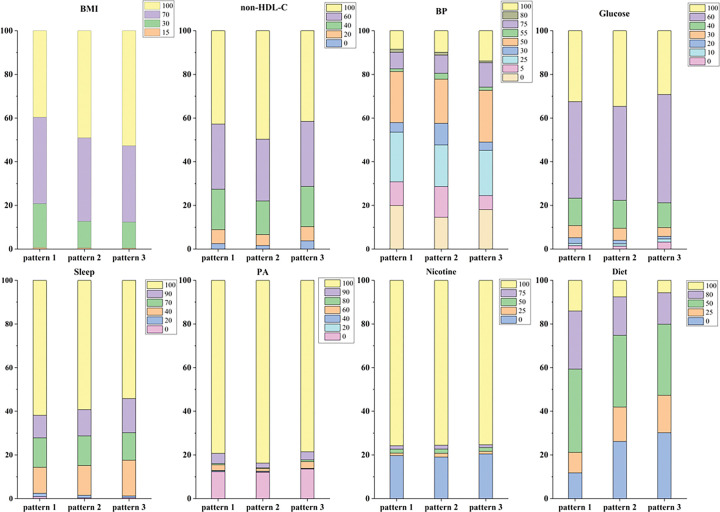
Distributions of absolute point scores for each CVH metric. Different colors represent the distribution of scores of each CVH metric under the three dietary patterns

As is shown in Fig. 2. 60%–80% of individuals had maximal scores (100) for sleep, physical activity and nicotine exposure, 40%–50% of individuals had highest level of BMI and non-HDL-C scores, and blood pressure and diet accounted for only about 10%. In different dietary patterns, the number of people with maximal scores in sleep and diet in pattern 1 was relatively high, non-HDL-C, blood glucose and physical activity in pattern 2 were relatively high, and BMI and blood pressure in pattern 3 were relatively high.

#### 3.2.3 Prevalence of CVH

Overall, 16.98% of individuals had high CVH, 77.30% had moderate CVH and 5.72% had low CVH. The proportion of high CVH in 60–69 years was higher than that in 70 year and above (pattern 2), female was higher than male in the three patterns and those with college or above education was higher than those with low education (pattern 3). No significant difference was found in income level and urban-rural distribution in CVH. In addition, there was no significant difference in the distribution of CVH status among the three patterns (p = 0.29). (Table 4).

#### 3.2.4 Distribution of ideal CVH metric and multivariate logistic analysis of CVH.

**Figure 3:**
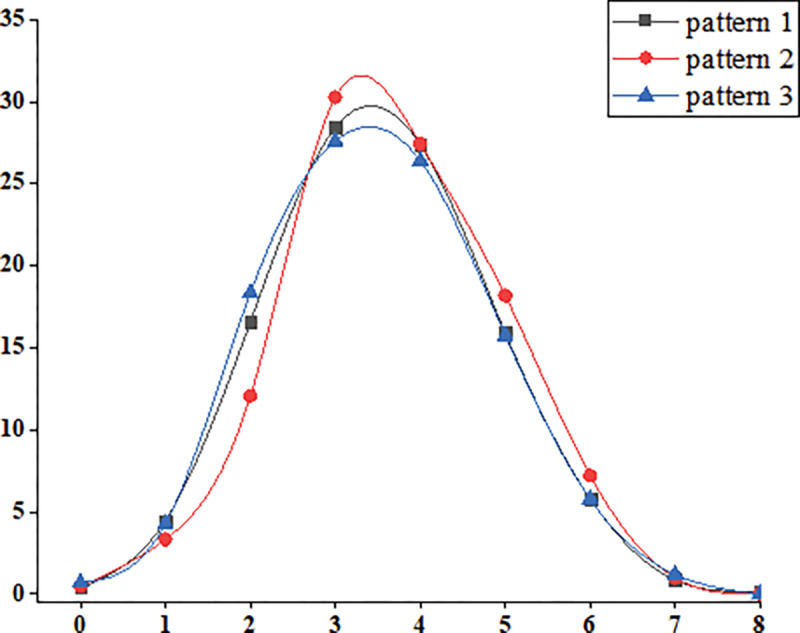
Distribution of ideal CVH metric. Black for Pattern 1; Red for pattern 2; Blue for Pattern 3.

There was an overall normal distribution of the number of ideal metrics. In three patterns, more than 50% of individuals had 3–4 ideal metrics, 6%–8% had ≥ 6 ideal metrics and 0.2% had 8 ideal metrics only in pattern 1. (Fig. 3)

Multinomial logit model showed the presence of 3–5 and 6–8 ideal metrics in pattern 2 was 1.45 times and 1.81 times of that in pattern 1, respectively. Compared with 60–69 years, the presence of 6–8 ideal metrics in the elderly aged 70 years and above was 35% lower (OR = 0.65,95%=0.49–0.87). Female who had 3–5 and 6–8 ideal metrics were 1.87 times and 3.42 times of men, respectively. The presence of 3–5 and 6–8 ideal metrics for those with college and above was 1.39 times and 1.99 times of those with primary or below, respectively. There were 6–8 ideal metrics in high income group, which were 31% lower than those in low income group (OR = 0.69,95%=0.47–1.00). (Table 5)

## 4. Discussion

In this study, three dietary patterns were obtained by clustering analysis. There were significant differences in some individual metric scores among the three patterns, but the overall CVH scores showed little difference. Most people had a moderate CVH status. Compared with the elderly men aged 70 years and above and those with lower education, the proportion of women, 60–69 years and those with college and above were higher in high CVH, and this subgroup and those in pattern 2 characterized by higher consumption of aquatic products, fruits and vegetables were likely to have more ideal metrics.

Lloyd-Jones et al. found that the total CVH score of Americans aged 65–79 years was 63.3, and physical activity score was the lowest [20]. In our study, the total CVH score of ≥ 60 years was 68.50, which was far lower than the highest score, and the blood pressure score was the lowest, and the number of people who had the highest score was only about 10%. Studies have found that hypertension has the strongest association with CVD, as the largest risk factor for CVD, accounting for 22.3% of its PAF [21].

Results from the CHNS showed that 71.3% of the elderly aged 60 years and above had elevated blood pressure (SBP ≥ 130mmHg and/or DBP ≥ 85mmHg). Therefore, it is necessary to pay attention to the pre-prevention of hypertension in the elderly to reduce the incidence of hypertension related diseases. In addition, the dietary score was relatively low, less than half of the highest score. Entering the aging stage, physical and mental function will decline to varying degrees, such as decreased chewing and digestion, delayed taste responses. Therefore, on the basis of a balanced diet for general adults, the elderly should be provided with a variety of foods that are high in energy and nutrient density and easy to digest and absorb, as well as animal foods and soy products that are rich in high quality protein. At the same time, the elderly should be encouraged to take active outdoor activities and maintain a healthy weight [22].

In the analysis of demographic characteristics, the overall CVH score ranged from 66.70 to 70.62, which may appear modest. But in the prior studies of LS7, Lee et al. followed up Korean adults aged 20–39 years for 16.1 years and found that the risk of cardiovascular events was reduced by 24%–42% for every 1 point increase in CVH score [23]. In the United States, a 31.9 year follow-up of adults aged 18–30 years found that for every 1 point increase, the risk of CVD and death was reduced by 27% and 31 %, respectively [24]. Thus, higher CVH scores were strongly associated with better health outcomes. In addition, among the eight metrics, 60%–80% of the elderly had the highest scores in sleep, physical activity and nicotine exposure, indicating that most people maintain good living habits, but smoking among the elderly still needs to be taken seriously. The report showed that the number of deaths caused by tobacco use in China accounted for nearly one third of the world, and tobacco use was far more harmful to the health of Chinese people than the global average, which further suggested the necessity of intervention and provided a basis for government departments to formulate tobacco control policies for special populations [3].

Our study found that the number of people with ≥ 6 ideal metrics in the three patterns was 6%–8%, 2% of people had the highest score in all 8 metrics only in pattern 1. In the United States, 4.1 % of the elderly had ≥ 5 and 0.7% have ≥6 of the 7 ideal metrics [25]. The prevalence of ideal CVH behaviors and factors in the elderly was alarming. Previous studies have shown that having a higher number of ideal CVH metrics could significantly reduce the risk of CVD morbidity and mortality. In American adults, compared with those with 0–1 ideal metrics, those with ≥ 6 ideal metrics had a 76% lower risk of death from CVD (HR = 0.24,95%CI = 0.13–0.47), and the risk showed a downward trend with the increase of the number of metrics [26]. Another meta-analysis of 9 prospective cohort studies involving 12 878 participants showed that having a higher number of ideal metrics was associated with 45% reduction in all-cause mortality, 75% in CVD mortality, 80% in CVD, and 69% in stroke [27]. Female had better CVH than male and were more likely to have more ideal metrics. Studies have shown that this difference was mainly attributed to the significantly higher smoking rate of males than females. If all smokers quit smoking, the ideal CVH rate would be increased by more than two times [28]. People with college and above had better CVH, and they may have better health awareness and be more likely to adopt health-related behaviors.

Analysis of different dietary patterns showed that people in pattern2 were more likely to have more ideal CVH metrics, and this pattern had higher intake of aquatic products, vegetables and fruits. The “Eastern healthy diet pattern”, represented by coastal areas such as Shanghai, Jiangsu and Zhejiang, was proposed for the first time in the Dietary Guidelines for Chinese residents. The main characteristics are diverse food, light and less oil, especially rich vegetables and fruits, fish and seafood, milk and beans [22]. Dietary guidelines for improving CVH proposed by the AHA that dietary patterns containing fish and seafood were associated with a lower risk of CVD [29], possibly due to the high omega-3 unsaturated fatty acids in fish [30]. In addition, the fish is softer, facilitating the digestion and absorption of the elderly. In the other two dietary patterns, the intake of aquatic products was only one-third of the recommended value. In addition, we should pay attention to the insufficient dairy intake, about 10% of the recommended amount in pattern 1 and pattern 2, and only 5% in pattern 3. Milk is an important source of dietary calcium and high quality protein, rich nutrition and easy to digest and absorb, the elderly should be instructed to choose suitable dairy products, and stick to use for a long time. According to the Burden of Disease Collaboration in the United States, low levels of each CVH metric led to substantial morbidity and mortality, and the main risk factor associated with the total burden of disease was an suboptimal diet [31]. Studies have shown that improving CVH score, especially dietary score, was of great significance to health [20].

In our study, “Life’s Essential 8” was used for the first time to evaluate the CVH status of Chinese elderly people based on differentiated regional dietary patterns, which was closely related to the LS7, but the “Life’s Essential 8” was more sensitive to individual differences. There are still some limitations in this study: 1) The 3d-24h dietary recalls may have recall bias and usually cannot assess the daily dietary intake. However, compared with the food consumption frequency collected by the food frequency method, the specific food intake collected by3d-24h was more accurate; 2) Our study only had lipid measurements and did not ask individuals if they were receiving lipid therapy, so the calculated scores could be high; 3) Nicotine exposure included e-cigarettes and electronic atomizer in addition to traditional cigarettes, but this study only collected traditional cigarettes, so the calculated score may be high. Therefore, caution should be exercised when comparing with similar results and extrapolating.

## 5. Conclusion

The elderly in China were generally in moderate CVH. Females, lower age, higher education and lower income groups had better CVH. Dietary patterns characterized by higher intake of aquatic products, vegetables and fruits were likely to have more ideal CVH metrics. It is necessary to take targeted intervention measures for key population and health factors with low scores to promote the improvement of cardiovascular health status.

## Figures and Tables

**Table 1:** Quantitative assessment of CVH metrics

Indicators		Quantification	

Health behaviors	BMI (kg/m^2^)	< 24	100
		24.0–27.9	70
		28.0–34.9	30
		35.0–39.9	15
		≥ 40.0	0
	
	Non-HDL-C (mg/dl)	< 130	100
		130–159	60
		160–189	40
		190–219	20
		≥ 220	0
	
	BP (mm/Hg)	< 120/80	100
		120–129/80	75
		130–139 or 80–89	50
		140–159 or 90–99	25
		≥ 160 or ≥ 100	0
		If drug-treated level, subtract 20 points	
	
	Blood glucose (mg/dl) or HbA1c (%)	No diabetes and FBG < 100(or HbA1c < 5.7)	100
		No diabetes and FBG:100–125 (or HbA1c: 5.7–6.4)	60
		Diabetes with HbA1c < 7.0	40
		Diabetes with HbA1c:7.0–7.9	30
		Diabetes with HbA1c:8.0–8.9	20
		Diabetes with HbA1c:9.0–9.9	10
		Diabetes with HbA1c:≥10.0	0

Health factors	Sleep (hours)	7– < 9	100
		9–<10	90
		6–<7	70
		5–<6 or ≥ 10	40
		4–<5	20
		< 4	0
	
	PA (min/week)	≥ 150	100
		120–149	90
		90–119	80
		60–89	60
		30–59	40
		1–29	20
		0	0
	
	Nicotine exposure	Never smoker	100
		Former smoker, quit ≥ 5y	75
		Former smoker, quit 1–<5y	50
		Former smoker, quit < 1y	25
		Current smoker	0
	
	Diet	≥ 95th percentile	100
		75th -94th percentile	80
		50th -74th percentile	50
		25th -49th percentile	25
		1th -24th percentile	0

BMI: body mass index; Non-HDL-C: non-high-density lipoprotein cholesterol; BP: blood pressure; PA: physical activity.

**Table 2:** Characteristics of different dietary patterns

	Pattern 1	Pattern 2	Pattern 3
Wheat	203.44^a^	89.13^b^	65.42^c^
Rice	125.33^c^	237.41^b^	291.90^a^
Other cereals	59.37^a^	17.28^b^	9.27^c^
Tubers	52.08^a^	26.90^c^	29.17^b^
Legumes	42.22^a^	34.93^a^	27.10^b^
Vegetables	232.35^b^	280.68^a^	267.44^a^
Fruits	55.31^a^	49.86^a^	26.60^b^
Livestock	40.15^c^	77.05^b^	105.18^a^
Poultry	5.20^b^	16.63^a^	18.03^a^
Fish and seafood	14.52^b^	50.57^a^	15.69^b^
Milk	39.32^a^	29.95^b^	16.53^c^
Eggs	36.29^a^	29.56^b^	16.48^c^
Nuts	5.19^a^	5.60^a^	2.73^b^
Cake	16.76^a^	11.97^a^	4.50^b^
Fungus	6.33^b^	8.77^a^	5.68^b^

a,b,c indicates that there was statistical significance between them if they have different letters, while the same letters or no letters indicated no statistical significance.

**Table 3:** CVH metric scores of different demographic characteristics ^#^

	BMI	non-HDL-C	BP	Glucose	Sleep	PA	Smoke	Diet	Total
Age									
60–69	72.36*	69.39	37.48*	67.61*	86.74*	88.82*	76.24*	55.40*	69.26*
70-	75.60	69.24	30.67	63.84	83.51	79.44	81.80	59.45	67.94
Gender									
male	74.41	73.85*	33.55	66.62	87.38*	81.32*	59.65*	56.82	66.70*
female	72.64	65.48	36.57	66.09	84.16	89.34	93.98	56.74	70.62
Education									
primary and above	73.85	67.93	32.74^c^	68.22^a^	83.07^b^	85.65	81.92^a^	59.49^a^	69.11
middle and high	72.46	70.76	36.04^b^	64.21^b^	87.05^a^	85.02	73.01^b^	54.76^b^	67.91^b^
college and above	75.18	69.95	41.11^a^	66.23	90.40^a^	87.49	80.53	53.42^b^	70.54^a^
Income									
low	75.20^a^	70.28	34.11^b^	68.15^a^	84.09^b^	83.18^b^	76.50	62.24^a^	69.22
middle	71.20^b^	69.21	34.20	67.28^a^	87.77^a^	86.03	78.77	56.77^b^	68.91
high	73.49	68.21	37.62^a^	62.90^b^	85.49	88.53^a^	79.63	49.44^c^	68.17
Area									
urban	74.91	70.02	39.16*	63.71*	86.44	87.36*	80.32	53.95*	69.48
rural	72.65	68.96	32.95	67.81	85.20	84.68	76.90	58.35	68.44
Dietary pattern									
Pattern 1	73.46^b^	69.34^b^	35.17^c^	66.34	85.64^a^	85.64^b^	78.13	56.77^a^	68.81^a^
Pattern 2	79.54^a^	73.81^a^	37.17^b^	67.68	85.24^a^	86.84^a^	78.28	42.04^b^	68.83^a^
Pattern 3	80.81^a^	68.07^b^	42.16^a^	65.08	83.77^b^	84.49^b^	77.47	37.80^c^	67.46^b^
Total	77.65	70.84	37.64	66.58	85.04	85.85	78.03	46.33	68.50

# The table only shows the CVH metric scores of demographic characteristics of pattern 1 (pattern 2 and 3 are shown in the Supplementary Table 2) and the total scores of the three patterns.

* p < 0.05

a,b,c indicates that there was statistical significance between them if they have different letters, while the same letters or no letters indicated no statistical significance.

**Table 4:** Proportion of CVH status in different demographic characteristics

	pattern 1				pattern 2				pattern 3			
	low	moderate	high	P-value	low	moderate	high	P-value	low	moderate	high	P-value
Age												
60–69	5.77	76.82	17.41	0.47	4.33	75.84	19.83	< 0.05	7.85	75.29	16.86	0.29
70-	6.02	79.03	14.95		5.86	80.03	14.11		5.43	78.52	16.05	
Gender												
male	8.97	78.77	12.25	< 0.05	8.37	79.93	11.70	< 0.05	11.26	76.41	12.34	< 0.05
female	3.18	76.53	20.29		1.79	75.25	22.96		3.14	76.75	20.11	
Education												
primary and above	5.11	76.85	18.04	0.16	5.67	76.39	17.95	0.67	5.24	79.05	15.71	< 0.05
middle and high	7.13	78.61	14.26		4.30	78.66	17.04		10.45	72.24	17.31	
college and above	4.52	76.88	18.59		3.91	78.26	17.83		3.90	76.62	19.48	
Income												
low	5.99	77.04	16.97	0.08	5.54	77.84	16.62	0.94	6.98	75.12	17.91	0.64
middle	3.61	79.19	17.20		4.53	77.90	17.57		5.96	77.98	16.06	
high	8.04	76.56	15.40		4.91	77.00	18.09		8.51	77.13	14.36	
Area												
urban	5.32	75.41	19.27	0.10	4.85	77.58	17.58	0.99	6.86	76.78	16.36	0.99
rural	6.15	78.77	15.08		4.98	77.42	17.61		6.88	76.48	16.64	
Total	5.86	77.57	16.58		4.93	77.48	17.60		6.87	76.59	16.53	

P _value_ <0.05 indicates that CVH distribution with different demographic characteristics is statistically significant.

**Table 5:** Multinomial logit model of ideal CVH status

Number of ideal CVH metrics			
	0–2	3–5	6–8
Dietary pattern			
Pattern 1	1.00	1.00	1.00
Pattern 2	1.00	1.45(1.20,1.75)*	1.81(1.33,2.47)*
Pattern 3	1.00	0.90(0.74,1.10)	0.96(0.68,1.37)
Age			
60–69	1.00	1.00	1.00
70-	1.00	1.06(0.90,1.24)	0.65(0.49,0.87)*
Gender			
male	1.00	1.00	1.00
female	1.00	1.87(1.59,2.19)*	3.42(2.57,4.54)*
Education			
primary and above	1.00	1.00	1.00
middle and high	1.00	1.05(0.88,1.26)	1.21(0.89,1.65)
college and above	1.00	1.39(1.04,1.87)*	1.99(1.24,3.19)*
Income			
low	1.00	1.00	1.00
middle	1.00	1.02(0.84,1.24)	0.96(0.69,1.33)
high	1.00	0.97(0.78,1.21)	0.69(0.47,1.00)*
Area			
urban	1.00	1.00	1.00
rural	1.00	0.86(0.72,1.03)	0.79(0.59,1.06)

* *p* < 0.05

“1.00” indicates that the group is a reference group.
